# Extracellular glypican‐1 affects tumor progression and prognosis in esophageal cancer

**DOI:** 10.1002/cam4.70212

**Published:** 2024-09-20

**Authors:** Rie Shibata, Hirotaka Konishi, Tomohiro Arita, Yusuke Yamamoto, Hayato Matsuda, Taiga Yamamoto, Takuma Ohashi, Hiroki Shimizu, Shuhei Komatsu, Atsushi Shiozaki, Takeshi Kubota, Hitoshi Fujiwara, Eigo Otsuji

**Affiliations:** ^1^ Division of Digestive Surgery, Department of Surgery Kyoto Prefectural University of Medicine Kyoto Japan

**Keywords:** esophageal cancer, glycocalyx, glypican‐1, GPC1, liquid biopsy

## Abstract

**Introduction:**

Cells are covered with a glycan surface layer that is referred to as the glycocalyx (GCX). It has been reported that the formation of the GCX is promoted on cancer cells and is associated with tumor growth and metastasis. Heparan sulfate proteoglycan glypican‐1 (GPC1) is a core protein of the GCX that is overexpressed in esophageal squamous cell carcinoma (ESCC) and is involved in the development and progression of cancer cells. The purpose of the present study is to analyze the utility of GPC1 as a new biomarker ralated to glycocalyx that reflects therapeutic effect and prognosis of ESCC.

**Methods:**

We measured the concentration of GPC1 protein in preoperative plasma from advanced esophageal cancer patients and examined its relationships with clinicopathological factors and therapeutic efficacy, and the effects of extracellular GPC1 were investigated.

**Results:**

The following clinical factors were significantly correlated with the preoperative high GPC1 concentration: male, tumor size ≥30 mm, venous invasion, pT factor ≥2, pStage ≥3, residual tumor, and distant metastatic recurrence. Both overall and recurrence‐free survival were significantly worse in the high GPC1 group. Extracellular GPC1 protein concentration reflected intracellular GPC1 expression. Furthermore, we examined the effects of extracellular recombinant human (rh)GPC1 on ESCC cells, and found that extracellular rhGPC1 affects cell motility, including migration and invasion.

**Conclusions:**

These results demonstrated the utility of extracellular GPC1 as a biomarker, which can be assayed from a less invasive blood sample‐based liquid biopsy. Extracellular GPC1 protein plays a role in both tumor cell motility and cancer progression. Thus, plasma GPC1 is a useful biomarker for esophageal cancer progression and may be a potential candidate of therapeutic target.

## Graphical abstract

This study presents novel findings on the role of glypican‐1 (GPC1) in esophageal squamous cell carcinoma (ESCC). We confirmed that high concentrations of GPC1 protein in preoperative plasma are significantly related to advanced stage and distant metastatic recurrence and was an independent poor prognostic factor in ESCC patients (*p* = 0.010, HR = 4.22). Furthermore, this research elucidates the role of extracellular GPC1 in enhancing cancer cell motility, thus contributing to tumor progression.

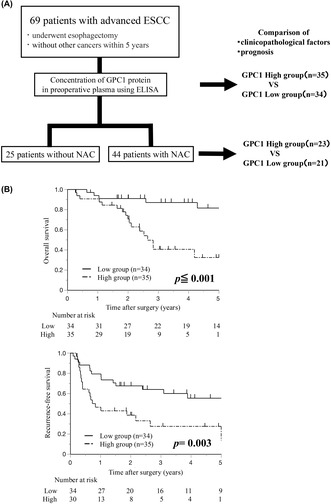

## 1 INTRODUCTION

Esophageal cancer is a gastrointestinal cancer with the poorest prognosis, and its morbidity and mortality are increasing worldwide.^1^, ^2^, ^3^, ^4^ Poor prognosis factors include a high frequency of lymph node metastasis and metastatic recurrence even after radical resection of the primary lesion.^5^, ^6^, ^7^, ^8^, ^9^, ^10^ Therefore, various treatment strategies for esophageal cancer have been developed, such as surgery, chemotherapy, and radiation therapy for primary or recurrent lesions.^11^, ^12^, ^13^ However, useful biomarkers to predict treatment efficacy and prognosis have not yet been established.^14^, ^15^ The development of new biomarkers and therapeutic strategies to diagnose and control metastasis is crucial for improving the prognosis of esophageal cancer.

The GCX is a glycoprotein complex that covers the surface of all cells in the human body and regulates various biological events.^16^, ^17^, ^18^ It plays a protective role in vascular endothelial cells, and a decrease in the GCX promotes the spread of inflammation.^19^, ^20^ Suppression of GCX expression also promotes the invasion and metastasis of cancer cells.^21^, ^22^, ^23^, ^24^ On the other hand, it has been reported that the GCX on the surface of cancer cells is overexpressed and thickened, which protects cancer cells and enhances their proliferation or metastatic potential.^21^, ^25^, ^26^, ^27^ In the present study, we focused on the GCX‐related molecules in cancer cells and aimed to develop new biomarkers that reflect esophageal cancer malignancy, therapeutic efficacy, or prognosis.

The GCX is composed of a core protein anchored to the cell membrane along with its binding glycans and lipids. The GCX is divided into the following four major classes: glycoproteins, glycolipids, mucins, and proteoglycans.^26^ We focused our studies on heparan sulfate proteoglycan glypican‐1 (GPC1; which is a core protein of the GCX) based on its expression profiles in various databases and previous reports.^28^, ^29^ GPC1 is a glycosylphosphatidylinositol (GPI) anchored protein located at the plasma membrane, which regulates the biological activities of the fibroblast growth factor (FGF) family, such as embryogenesis, cell growth, morphogenesis, tissue repair, and tumor growth.^30^, ^31^, ^32^ It has been reported that GPC1 is overexpressed in ESCC and is involved in the development and progression of cancer cells.^33^, ^34^, ^35^ On the other hand, in various cancer studies, a secreted form of GPC1 has been reported to play additional roles in cancer biology. In pancreatic and prostate cancer, circulating GPC1 protein has been shown to be a prognosis biomarker.^36^, ^37^, ^38^ However, there are no reports which have investigated GPC1 in the plasma of esophageal cancer patients using liquid biopsy.

In the present study, we measured the concentration of GPC1 protein in the plasma of esophageal cancer patients. We also examined its relationships with clinicopathological factors and therapeutic efficacy. Lastly, the effects of rhGPC1 protein on esophageal cancer cell function was also examined.

## 2 MATERIALS AND METHODS

### 2.1 Patients and clinical samples

Preoperative plasma samples were obtained from 69 advanced ESCC patients expected for radical esophagectomy (clinical stages II–IV). All patients underwent esophagectomy with lymphadenectomy at Kyoto Prefectural University of Medicine Hospital (Kyoto, Japan) between March 2014 and December 2021. Patients with a previous history of other cancers within the past 5 years were excluded. Table 1 shows patient characteristics. The patients were diagnosed with pathological stages I–IV after surgery. Clinicopathological findings and postoperative courses were gathered from medical records and databases. Tumor staging was classified according to the 8th edition of the Union for International Cancer Control (UICC)/tumors, nodes, and metastases staging system.^39^ The clinical and histological effects of preoperative treatment were classified according to the Japanese Classification of Esophageal Cancer, 11th edition.^40^, ^41^

**TABLE 1:** Patient characteristics.

Variables		*n* = 69
Age (years)	Median (min–max)	68 (52–86)
Sex	Male/female	59/10
Body mass index (kg/m^2^)	Median (min–max)	20.8 (13.38–28.6)
PS^a^	0/1/2	47 (68%)/17 (25%)/5 (7%)
Neoadjuvant chemotherapy	+/−	44 (64%)/25 (36%)
Plasma GPC1 concentration (ng/mL)	Median (min–max)	4.67 (2.12–35.75)
Surgical approach	Thoracotomy/mediastinoscopy	13 (19%)/56 (81%)
Surgical time (min)	Median (min–max)	340 (166–197)
Blood loss (mL)	Median (min–max)	164 (18–1500)
Tumor location	Ut/Mt/Lt	10 (14%)/33 (48%)/26 (38%)
Differentiation	Well/moderate/poor	31 (45%)/29 (42%)/9 (13%)
Pathological T factor^b^	T0/1/2/3/4	8 (11%)/19 (28%)/8 (11%)/29 (42%)/5 (7%)
Pathological N factor^b^	N0/1/2/3	23 (33%)/28 (41%)/12 (17%)/6 (9%)
Pathological M factor^b^	M0/1 (LYM)	66 (96%)/3 (4%)
Pathological stage^b^	Stage 0/I/II/III/IV	7 (10%)/9 (13%)/19 (28%)/29 (42%)/5 (7%)
Residual tumor	R0/1/2	64 (93%)/1 (1%)/4 (6%)
Recurrence^c^	+/−	34 (53%)/30 (47%)

^a^ According to the Eastern Cooperative Oncology Group (ECOG) Performance Status.

^b^ According to the 8th edition of the Union for International Cancer Control tumor, node, metastasis classification system.

^c^ R1 and R2 cases were excluded from this analysis.

The plasma was extracted from the collected blood samples with a three‐spin protocol (i.e., 350 × *g* at 4°C for 30 min, followed by 700× *g* at 4°C for 5 min, and 1600× *g* at 4°C for 5 min) to ensure the removal of residual cellular components. The resulting plasma samples were stored at −80°C. Plasma samples of 60 healthy volunteers (HV) were collected before surgery for noncancerous diseases and processed with the same procedures.

This study was conducted in accordance with the principles of the Declaration of Helsinki, and all patients provided written informed consent before surgery.

### 2.2 Cell culture

The human ESCC cell lines TE2, TE5, TE8, and TE11 were obtained from the RIKEN BioResource Center Cell Bank (Ibaraki, Japan). The human ESCC cell lines KYSE70, KYSE150, and KYSE170 were obtained from the Japanese Collection of Research Bioresources Cell Bank (Osaka, Japan). The human normal esophageal squamous epithelial cell line Het‐1A was obtained from the American Type Culture Collection (Rockville, MD, USA). All cell lines were maintained in Roswell Park Memorial Institute (RPMI) medium (Nakalai Tisque, Kyoto, Japan) supplemented with 10% fetal bovine serum (FBS; System Biosciences, Palo Alto, CA, USA). The cells were incubated in a humidified 37°C incubator with 5% carbon dioxide.

### 2.3 Collection of cell culture medium

Het‐1A, TE11, and KYSE170 cells were seeded in 24‐well plates at 1.0 × 10^4^ cells/well. The culture medium was collected 48 h after seeding, processed with a three‐spin protocol (see above), and stored at −80°C.

### 2.4 Cis‐diamminedichloro‐platinum (II) (CDDP) treatments and collection of culture medium

TE2 and KYSE70 cells were seeded in 24‐well plates at 2.0 × 10^4^ cells/well. After 24 h, the culture medium was discarded, and the cells were incubated for 48 h in fresh culture medium in the presence of graded concentrations of CDDP (0, 4, 8, and 16 μmol/L). Next, the medium was collected using the procedure mentioned above, and the GPC1 protein concentration in the medium was measured by ELISA (see below).

The viable cells at the time of medium collection were assessed by a colorimetric water‐soluble tetrazolium salt assay (Cell Counting Kit‐8; Dojindo Laboratory, Kumamoto, Japan) and the absorbance at 450 nm was measured. Each ELISA measurement was normalized to the results of the viability assay.

### 2.5 Measurement of the GPC1 protein concentration by an enzyme‐linked immunosorbent assay (ELISA)

Extracellular GPC1 protein concentration in plasma and in the culture medium was measured using a Human Glypican1 ELISA Kit (Ray Biotech, Tokyo, Japan) according to the instructions provided by the manufacturer. All 69 ESCC patients were divided into two groups (low and high GPC1 groups), based on a median ELISA measurement of 4.67 ng/mL.

### 2.6 RNA extraction and quantification of mRNA expression

Total RNA was extracted from the cultured cells using a miRNeasy Mini kit (Qiagen, Hamburg, Germany), according to the manufacturer's instructions. The reverse transcription reaction was performed using a High‐Capacity cDNA Reverse Transcription Kit (Applied Biosystems, Foster City, CA, USA) as per the manufacturer's instructions (25°C for 10 min followed by 37°C for 120 min and 85°C for 5 min). The level of mRNA expression was measured using the following TaqMan Gene Expression Assays: GPC1 (Assay ID: Hs00892476) and β‐actin (Assay ID: Hs99999903_m1) (Applied Biosystemsm, Foster City, CA, USA), according to the manufacturer's instructions. Quantitative reverse transcription‐pathological complete response (qRT‐PCR) was performed using a StepOnePlus PCR System and the cycle threshold (Ct) value was calculated using StepOne Software v2.3 (Applied Biosystems, Foster City, CA, USA). The thermocycling conditions were 95°C for 10 min, followed by 40 cycles at 95°C for 15 s, 55°C for 30 s, and 72°C for 30 s. The results were evaluated using the 2^−ΔΔCt^ method relative to the expression of β‐actin.^42^

### 2.7 Western blotting analysis

Proteins were extracted using the Mammalian Protein Extraction Reagent (Thermo Fisher Scientific, Waltham, MA, USA). The protein concentration was measured using the Protein Assay Rapid Kit wako II (Wako, Tokyo, Japan). Each protein sample (22 μg) was separated by 10% sodium dodecyl sulfate‐polyacrylamide gel electrophoresis and subsequently transferred onto polyvinylidene difluoride membranes (GE Healthcare, Chicago, IL, USA). The membranes were blocked for 1 h at room temperature with Tris‐buffered saline containing 0.05% Tween 20 (TBST) and 5% bovine serum albumin (Sigma‐Aldrich, St Louis, MO, USA). Next, the membranes were incubated with the following primary antibodies at 4°C overnight: anti‐Glypican1 (1:2000 dilution, anti‐mouse, MAB2600; Sigma‐Aldrich, St Louis, MO, USA) and anti‐β‐actin antibody (1:20000 dilution, anti‐mouse, no. A2228‐200UL; Sigma‐Aldrich, St Louis, MO, USA). The membranes were meticulously washed with TBST to eliminate any excess primary antibodies, followed by incubation with anti‐rabbit (no. 7074S) or anti‐mouse (no. 7076S) secondary antibodies (both from Cell Signaling Technology, Danvers, MA, USA) for 1 h at room temperature. The target proteins were detected using the ECL Plus Western Blotting Detection System (GE Healthcare, Chicago, IL, USA). Candidate bands obtained by Western blot (WB) were quantified using the image analysis software ImageJ.

### 2.8 Extracellular rhGPC1 reagent

The rhGPC1 protein was obtained from Bio‐Techne (R&D Systems, no. 4519‐GP, Minneapolis, MN, USA). The reagent was used in each experiment at a concentration of 200 ng/mL and was added directly to the culture medium. The alterations in intracellular GPC1 expression induced by extracellular rhGPC1 treatment were confirmed by qRT‐PCR and WB 48 h after treatment.

### 2.9 Proliferation assay

TE5 and KYSE170 cells were seeded at 1.0 × 10^4^ cells/well in 24‐well plates. After 24 h, the medium was replaced and rhGPC1 was added. The viable cells were evaluated by a colorimetric water‐soluble tetrazolium salt assay (Cell Counting Kit‐8; Dojindo Laboratories, Kumamoto, Japan) and the absorbance at 450 nm was measured. Cell proliferation was assessed 0, 24, 48, and 72 h after addition of rhGPC1.

### 2.10 Migration and invasion assays

The BD BioCoat MatrigelTM Invasion Chamber kit (BD Biosciences, Franklin Lakes, NJ, USA) was used for migration and invasion assays. The upper surface of the 6.4‐mm filter with 8‐μm pores is coated with matrigel for the invasion assay, but it is not coated for the migration assay. To evaluate the effects of extracellular GPC1, we compared the migration and invasion abilities of TE5 (4.0 × 10^5^ cells/well) and KYSE170 (1.0 × 10^5^ cells/well) cells with or without rhGPC1 treatment (200 ng/mL). The cells were seeded in the upper Boyden chamber containing RPMI without FBS, and the lower chamber was filled with RPMI with FBS. After a 48‐h incubation at 37°C, the cells that migrated or infiltrated to the lower membrane were fixed and stained with the Diff‐Quik Stain Kit (Sysmex, Kobe, Japan). The number of stained cells at six random fields was counted directly using a microscope, and the mean value was compared. Each assay was conducted in triplicate.

### 2.11 Drug susceptibility test

TE5 (6 × 10^3^ cells/well) and KYSE170 cells (3 × 10^3^ cells/well) were seeded in 96‐well plates and incubated for 24 h. The cells were treated with 200 ng/mL of rhGPC1 for 6 h, then treated with 80 μmol/L of CDDP for 48 h. The susceptibility to CDDP was investigated by cell viability assays, which were measured with a colorimetric water‐soluble tetrazolium salt assay (Cell Counting Kit‐8; Dojindo Laboratories, Kumamoto, Japan).

### 2.12 Statistical analysis

All statistical analyses were performed using the statistical software JMP Pro® 17.0.0 (2016 SAS Institute Inc., Cary, NC, USA). Differences between paired or unpaired samples were compared using the Wilcoxon signed‐rank test or Student's *t*‐test, respectively. Categorical variables in two groups were compared using the chi‐squared test. Survival curves for recurrence‐free survival (RFS) and overall survival (OS) were calculated and plotted using the Kaplan–Meier method and compared using the log‐rank test. Multivariate survival analysis was performed using the Cox proportional hazard regression model. A *p*‐value of less than 0.05 was considered significant.

## 3 RESULTS

### 3.1 Relationships between the GPC1 protein concentration in preoperative plasma and clinical characteristics

**FIGURE 1:**
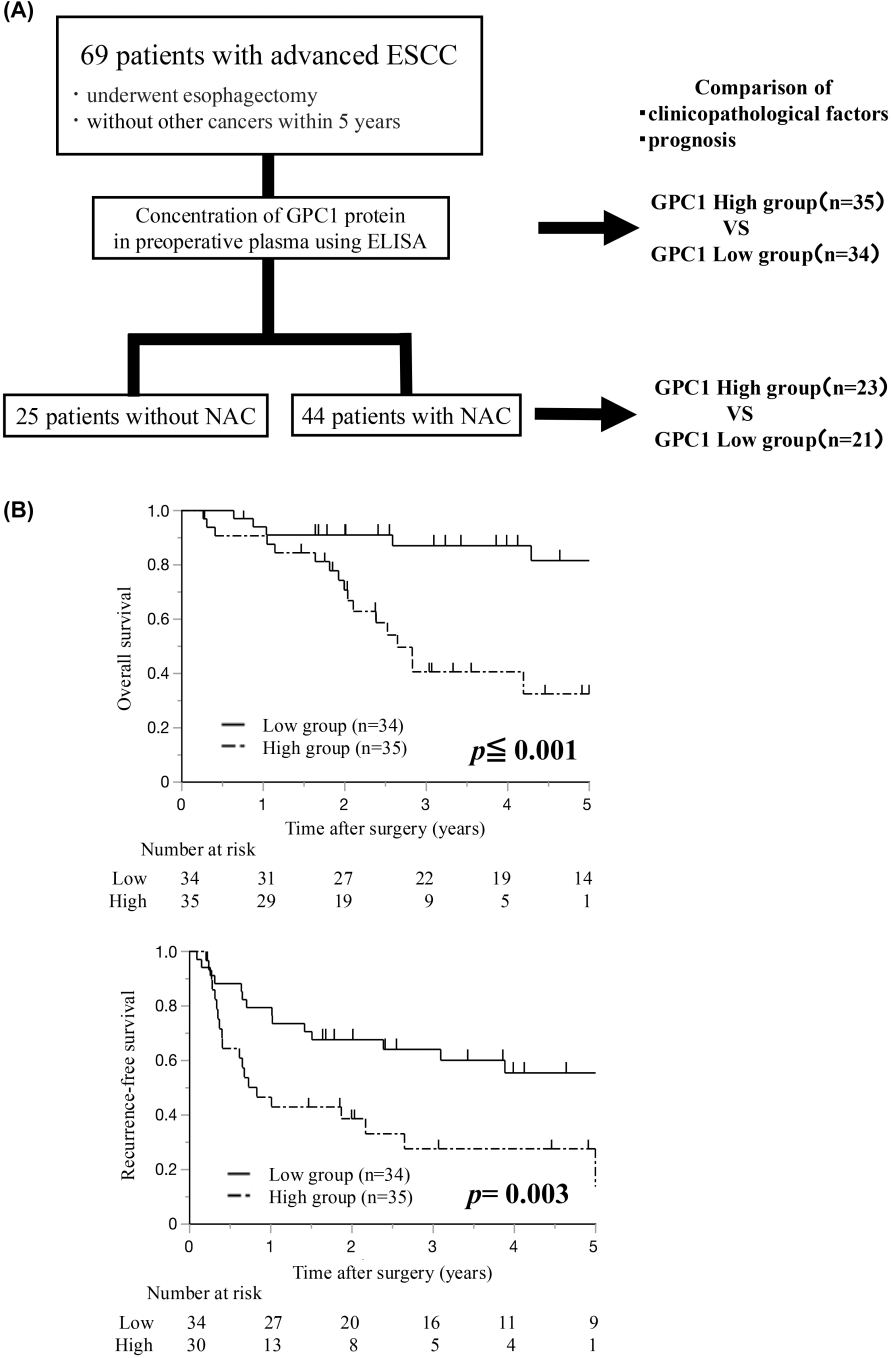
(A) Schematic diagram of the present study. (B) Kaplan–Meier curves for OS and RFS after surgery in all patients. The patients were divided into high and low groups according to the median value of the GPC1 protein concentration. GPC1, glypican‐1; OS, overall survival; RFS, recurrence‐free survival.

We measured preoperative plasma GPC1 protein concentrations by ELISA in 69 patients who underwent esophagectomy for advanced esophageal cancer. The relationships between GPC1 protein levels and clinicopathological characteristics were analyzed. Table 1 presents the patient characteristics of all 69 patients, and a schematic diagram is shown in Figure 1A. Patients were divided into high and low groups by the median value of the GPC1 protein concentration (4.67 ng/mL) according to the distribution of plasma GPC1 concentration (Figure S1A).

As shown in Table 2, the following clinical factors were significantly correlated with the high GPC1 levels group: male (*p* = 0.036), tumor size ≥30 mm (*p* = 0.028), presence of venous invasion (*p* = 0.041), pT factor ≥2 (*p* = 0.005), pStage ≥3 (*p* = 0.022), residual tumor (*p* = 0.022), and distant metastatic recurrence (*p* = 0.022).

**TABLE 2:** Relationships between preoperative GPC1 concentration in plasma and clinicopathological factors.

Variables	*n* = 69	Plasma GPC1 concentration				*p‐*value^a^
		Low group (*n* = 34) (≤ 4.67 ng/mL)		High group (*n* = 35) (>4.67 ng/mL)		
Age (years)						
≥65	45	22	(49%)	23	(51%)	0.930
<65	24	12	(50%)	12	(50%)	
Sex						
Male	59	26	(44%)	33	(56%)	**0.036**
Female	10	8	(80%)	2	(20%)	
Body mass index (kg/m^2^)						
≥21	33	13	(39%)	20	(61%)	0.116
<21	36	21	(58%)	15	(42%)	
PS^b^						
1,2	22	11	(50%)	11	(50%)	0.934
0	47	23	(49%)	24	(51%)	
Neo adjuvant chemotherapy						
+	44	21	(48%)	23	(52%)	0.733
−	25	13	(52%)	12	(48%)	
Location						
Ut	10	4	(40%)	6	(60%)	0.666
Mt	33	18	(55%)	15	(45%)	
Lt	26	12	(46%)	14	(54%)	
Differentiation						
Well	31	17	(55%)	14	(45%)	0.706
Moderate	29	13	(45%)	16	(55%)	
Poor	9	4	(44%)	5	(56%)	
Tumor size (mm)						
≥30	43	14	(33%)	29	(67%)	**0.028**
<30	26	20	(77%)	6	(23%)	
Lymphatic invasion^c^						
Presence	29	11	(38%)	18	(62%)	0.109
Absence	40	23	(58%)	17	(42%)	
Venous invasion^c^						
Presence	39	15	(38%)	24	(62%)	**0.041**
Absence	30	19	(63%)	11	(37%)	
pT factor^c^						
T2, 3, 4	42	15	(36%)	27	(64%)	**0.005**
T0, 1	27	19	(70%)	8	(30%)	
pN factor^c^						
N1, 2, 3	46	19	(41%)	27	(59%)	0.061
N0	23	15	(65%)	8	(35%)	
pStage^c^						
3, 4	34	12	(35%)	22	(65%)	**0.022**
0, 1, 2	35	22	(63%)	13	(37%)	
Residual tumor						
R1, 2	5	0	(0%)	5	(100%)	**0.022**
R0	64	34	(53%)	30	(47%)	
Recurrence^d^						
+	34	15	(44%)	19	(56%)	0.124
−	30	19	(63%)	11	(37%)	
Distant metastasis^d^						
+	17	5	(29%)	12	(61%)	**0.022**
−	47	29	(62%)	18	(38%)	

^a^ *p‐*values were calculated using the chi‐squared test.

^b^ According to the Eastern Cooperative Oncology Group (ECOG) performance status.

^c^ According to the 8th edition of the Union for International Cancer Control tumor, node, metastasis classification system.

^d^ R1 and R2 cases were excluded from this analysis.

### 3.2 Influence of the GPC1 concentration on survival

Both overall and recurrence‐free survival were significantly worse in the high GPC1 group (*p <* 0.001 and *p* = 0.003, respectively, Figure 1B). Univariate analysis indicated that the residual tumor (*p* < 0.001), tumor size ≥30 mm (*p* = 0.027), lymphatic invasion (*p* = 0.007), pT factor ≥2 (*p* = 0.046), pN factor ≥2 (*p* < 0.001), pStage ≥3 (*p* = 0.012), and high GPC1 concentration (*p* < 0.001) were correlated with poor overall survival (Table 3). Moreover, in multivariate analysis, only high GPC1 concentration was an independent prognostic factor (HR 4.22, 95% CI 1.41–12.57, *p* = 0.010).

**TABLE 3:** Survival analysis in univariate and multivariate analyses.

Variables	*n* = 69	Univariate		Multivariate		
		OS (%)	*p* value^a^	HR	95% C.I.	*p*‐value^b^
Age (years)						
≥65	45	73.3	0.052			
<65	24	50.0				
Sex						
Male	59	64.4	0.767			
Female	10	70.0				
PS^c^						
1, 2	22	77.3	0.254			
0	47	59.6				
NAC						
+	44	61.4	0.549			
−	25	72.0				
Operation time (min)						
≥340	37	62.2	0.884			
<340	32	68.8				
Blood loss (mL)						
≥160	34	67.6	0.209			
<160	35	62.9				
Residual tumor						
R 1,2	5	0.0	**<0.001**	2.84	0.76–10.62	0.121
R 0	64	70.3				
Differentiation						
Moderate/poor	38	60.5	0.213			
Well	31	71.0				
Tumor size (mm)						
≥30	49	59.2	**0.027**	1.79	0.45–7.07	0.408
<30	20	80.0				
Lymphatic invasion^d^						
Presence	29	51.7	**0.007**	1.41	0.50–3.87	0.499
Absence	40	75.0				
Vascular invasion^d^						
Presence	39	61.5	0.098			
Absence	30	70.0				
pT factor^d^						
T 2–4	42	57.1	**0.046**	1.31	0.42–4.05	0.639
T 0–1	27	77.8				
pN factor^d^						
N 2–3	18	33.3	**<0.001**	2.03	0.73–5.65	0.173
N 0–1	51	76.5				
pStage^d^						
Stages 3–4	34	52.9	**0.012**	−	−	−
Stages 0–2	35	77.1				
Plasma GPC1 concentration						
≥4.67	35	48.6	**<0.001**	4.22	1.41–12.57	**0.010**
<4.67	34	82.4				

*Note*: Bold values indicate significant differences (*p* value <0.05).

Abbreviations: HR, hazard ratio; NAC, neoadjuvant chemotherapy; OS, overall survival; 95% CI, 95% confidence interval.

^a^ *p* value was calculated using the log‐rank test.

^b^ *p* value was calculated using the Cox hazard model.

^c^ According to the Eastern Cooperative Oncology Group (ECOG) Performance Status.

^d^ According to the 8th edition of the Union for International Cancer Control tumor, node, metastasis classification system.

In 44 patients with NAC, the results of correlation analysis between plasma GPC1 concentration and clinical characteristics were similar to those obtained from all patients (Table S1). In terms of the chemotherapy effects such as efficacy or adverse events, high histological effects (Grade 2/3) were correlated with low preoperative plasma GPC1 concentration (*p* = 0.035, Table S2), and pCR by NAC was frequently observed in the low GPC1 group (*p* = 0.028, Table S2). The overall and recurrence‐free survival rates were also significantly worse in the high GPC1 group (*p* < 0.001 and *p* = 0.001, respectively, Figure S1B).

### 3.3 GPC1 levels in ESCC cell lines and culture medium

**FIGURE 2:**
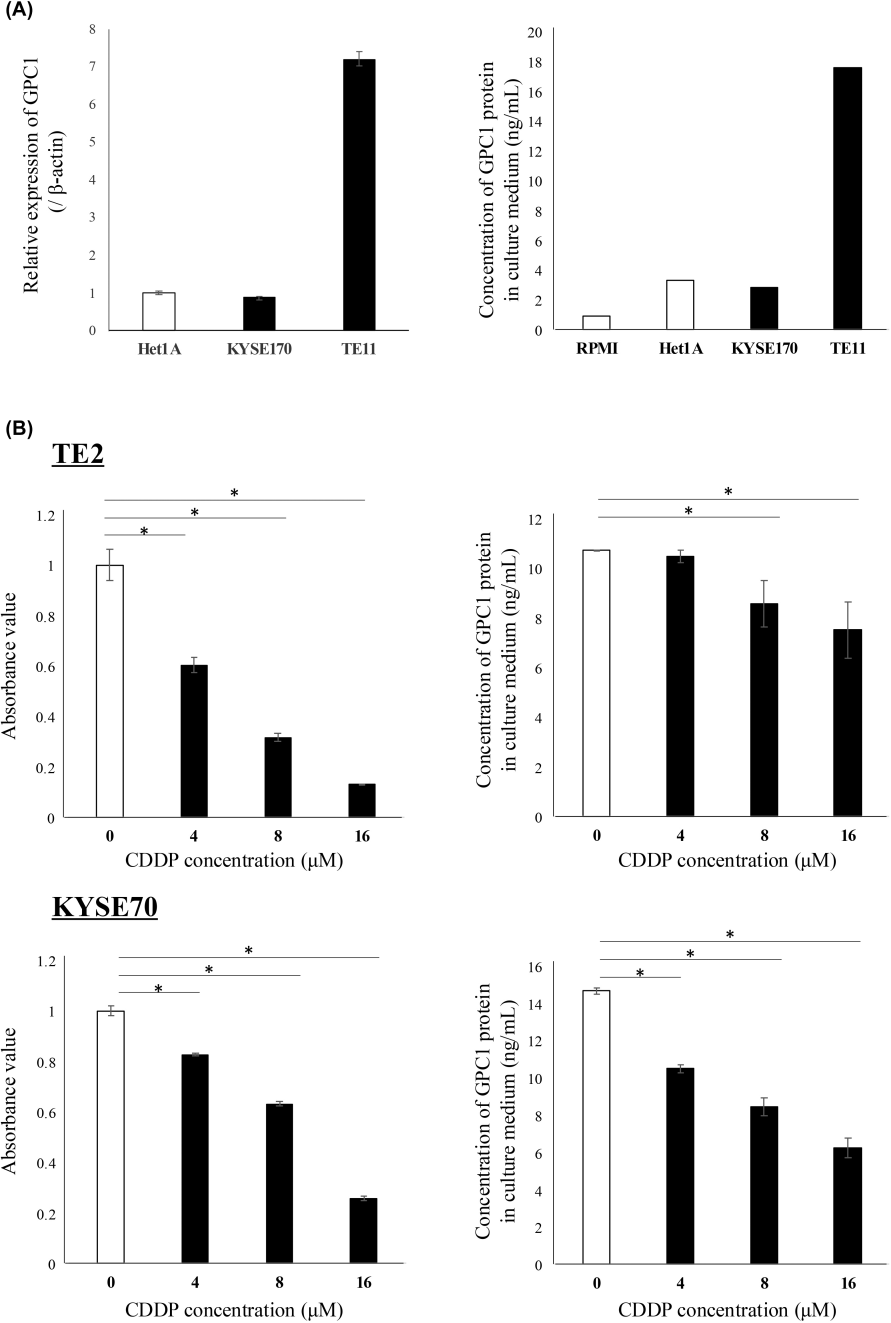
GPC1 expression in ESCC cell lines and GPC1 protein concentration in the culture medium of ESCC cell lines. (A) GPC1 expression in ESCC cell lines (KYSE170 and TE11) as well as a normal esophageal epithelial cell line (Het‐1A) and GPC1 protein concentration in the medium. (B) Evaluation of ESCC cell lines: Comparison of the viable cells and GPC1 protein concentration in the medium at each CDDP concentration. The data were analyzed with the Mann–Whitney *U*‐test (*p < 0.05). All experiments were performed in triplicate. CDDP, cis‐diamminedichloro‐platinum; ESCC, esophageal squamous cell carcinoma; GPC1, glypican‐1.

GPC1 expression was evaluated in several ESCC cell lines (Figure S3), and suitable cell lines were selected for each experiment based on the GPC1 expression profile. GPC1 expression and protein concentration in the medium were evaluated in KYSE170 and TE11 cell lines, which exhibit low and high GPC1 expression, respectively. We also determined the GPC1 expression and protein concentration in the normal esophageal epithelial cell line, Het‐1A (Figure 2A). The GPC1 protein concentration in the culture medium was high in cell lines with higher GPC1 expression. On the other hand, the GPC1 protein concentration in the culture medium decreased as the number of TE2 and KYSE70 cells was decreased due to the CDDP concentration (Figure 2B).

### 3.4 Effects of extracellular rhGPC1 on ESCC cells

**FIGURE 3:**
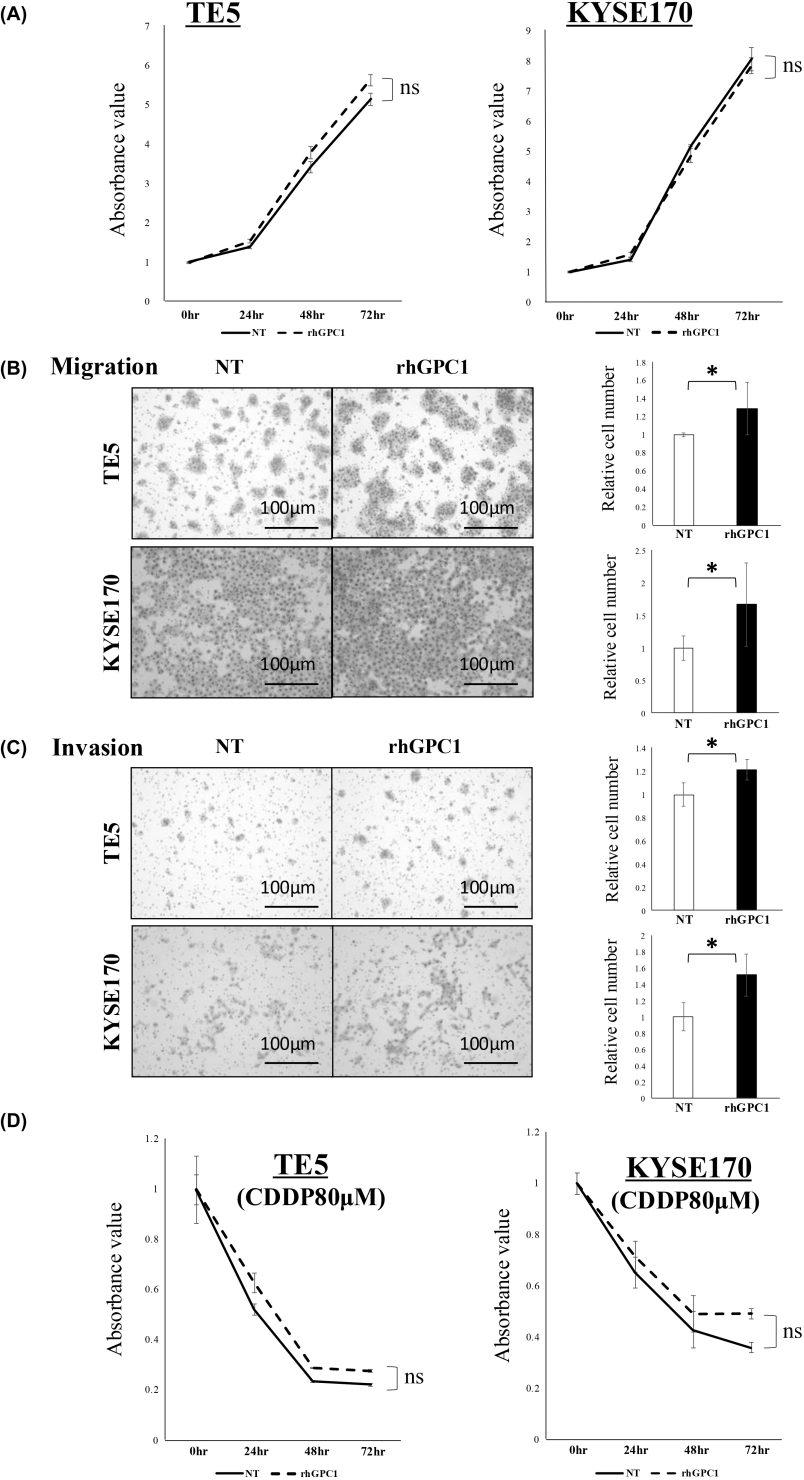
The effects of extracellular rhGPC1 on ESCC cells. (A) The effects of rhGPC1 (200 ng/mL) on cell proliferation was evaluated in TE5 and KYSE170 cells. The data represent the mean absorbance value ± SD. (B and C) The effects of rhGPC1 (200 ng/mL) on (B) migration and (C) invasion of TE5 and KYSE170 cells were evaluated using the transwell migration and invasion assay, respectively. The data represent the mean ± SD of relative cell numbers (*p < 0.05). (D) The changes in susceptibility of TE5 and KYSE170 cells to CDDP (80 μmol/L) were evaluated after the addition of rhGPC1 (200 ng/mL). The data represent the mean absorbance value ± SD. All experiments were performed in triplicate. CDDP, cis‐diamminedichloro‐platinum; ESCC, esophageal squamous cell carcinoma; rhGPC1, recombinant human glypican‐1; SD, standard deviation.

**FIGURE 4:**
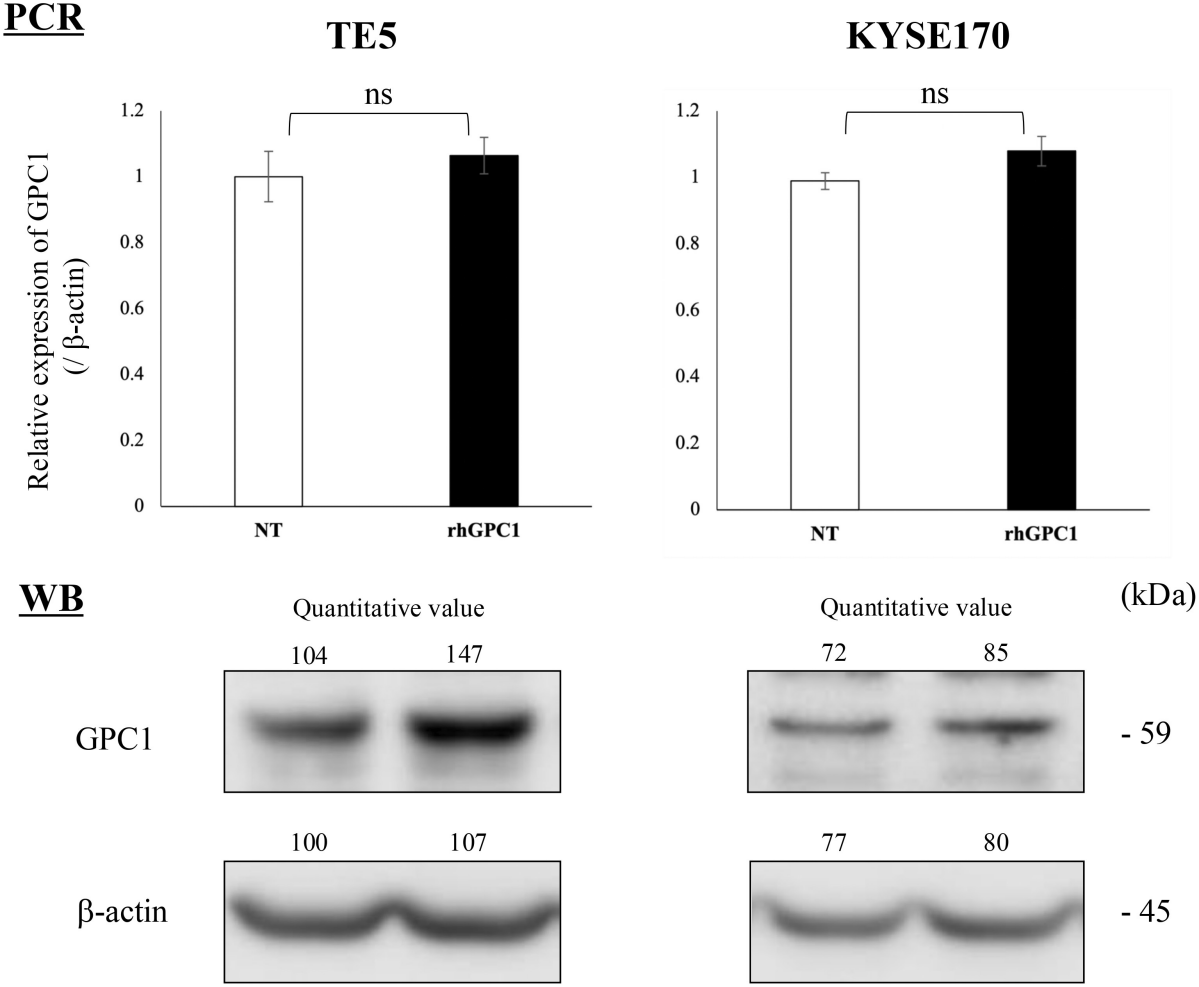
The alterations in GPC1 mRNA and protein levels after extracellular rhGPC1 treatment were analyzed using qRT‐PCR and WB, respectively. Quantitative values obtained by ImageJ analysis are shown. GPC1, glypican‐1; qRT‐PCR, quantitative reverse transcription‐pathological complete response; rhGPC1, recombinant human glypican‐1; WB, Western blotting.

The proliferation ability of TE5 and KYSE120 cells, which are cell lines with lower GPC1 expression (Figure S3), was not changed by the addition of extracellular rhGPC1 (200 ng/mL) (Figure 3A). In contrast, both migration and invasion were markedly promoted by the addition of extracellular rhGPC1 (Figure 3B,C). In the drug susceptibility assay, resistance to CDDP slightly increased with the addition of extracellular rhGPC1, but it was not a significant difference (Figure 3D). Even though no changes in GPC1 expression in ESCC cell lines were observed after the addition of extracellular rhGPC1, GPC1 protein expression in ESCC cell lines was upregulated as analyzed by WB (Figure 4).

## 4 DISCUSSION

GPC1, one of the GCX constituent molecules, plays a critical role in intercellular communication and signaling, which affects various cellular processes such as growth, differentiation, and migration.^28^, ^31^, ^43^ GPC1 also contributes to the protection against cell dysfunction and vascular disease in endothelial cells.^44^ GPC1 expression was increased in hepatocellular carcinoma, breast cancer, cervical cancer, prostate cancer, and glioma.^45^, ^46^, ^47^, ^48^, ^49^ Moreover, expression of GPC1 promotes cancer growth and metastasis. In pancreatic cancer, it has been reported that regulation of GPC1 expression affects cell functions such as cell signaling, tumor growth, angiogenesis, and metastasis.^50^, ^51^ In the present study, we analyzed the association between GPC1 protein levels in plasma and clinicopathological factors as well as prognosis in esophageal cancer patients. Furthermore, the effect of extracellular GPC1 protein on esophageal cancer cells was evaluated.

Several studies have reported the utility of GPC1 as a biomarker of ESCC. Hara et al. investigated the correlation between GPC1 expression and chemotherapy resistance as well as prognosis in ESCC.^33^ GPC1 was highly expressed in ESCC, and patients with high GPC1 expression had poor prognosis due to CDDP resistance. Li et al. similarly reported that GPC1 was overexpressed in ESCC tissues and correlated with poor prognosis.^35^ They also found that GPC1 promotes cell proliferation and motility by influencing the phosphatase and tensin homolog (PTEN)/Akt/β‐catenin signaling pathway. Although the roles of GPC1 expression in ESCC has been reported previously, this study is the first to evaluate the effects of extracellular GPC1 in esophageal cancer.

In the present study, tumor size, venous invasion, stage progression, residual tumor, and distant metastatic recurrence were associated with high GPC1 protein concentration in preoperative plasma (Table 2). Furthermore, a high GPC1 concentration was an independent factor for poor prognosis (Figure 1B and Table 3). In patients with neoadjuvant chemotherapy (NAC), a high GPC1 concentration in plasma was correlated with a lower histologic response (Grade 1) to chemotherapy (Table S2). Moreover, low GPC1 concentration in plasma was significantly correlated with pathological CR (Table S2). These results are consistent with previous reports demonstrating the roles of GPC1 in ESCC, and furthermore, reveal the potential of extracellular GPC1 as a biomarker, which can be analyzed as a less invasive blood sample‐based liquid biopsy. The ability to identify patients at high risk of metastatic recurrence after surgery may be important for considering the need for further surgery, adjuvant chemotherapy, and careful postoperative follow‐up.

We also analyzed plasma GPC1 levels in healthy volunteers (*n* = 60) and esophageal cancer patients (*n* = 39). The plasma samples were collected before any treatments such as chemotherapy or surgery, but there was no significant difference in plasma GPC1 levels (Figure S2A). We think these results may be due to the fact that plasma GPC1 concentration is affected not only by the tumor but also by vascular endothelial damage or other host factors. GPC1 is present in endothelial cells throughout the body as a glycocalyx that protects normal tissues, and previous reports have shown that GPC1 expression is suppressed by atherosclerosis.^44^ Plasma GPC1 concentration will be also affected by GPC1 expression in such normal tissues. Esophageal cancer patients have more risk factors for atherosclerosis, such as older age, smoking, and hypertension, compared to healthy volunteers. Therefore, plasma GPC1 derived from normal endothelial cells of esophageal cancer patients will be lower than that of healthy volunteers, and difference in blood GPC1 concentration between healthy volunteer and esophageal cancer patients may be unclear. If the backgrounds of healthy volunteers could be matched with those of esophageal cancer patients, the differences might be confirmed, but it is currently difficult to collect such control samples.

In addition, no consistent trend was observed in the change of plasma GPC1 level before and after surgery (Figure S2B). Past reports have suggested that systemic inflammation such as sepsis, and invasive procedures such as surgery or trauma, can result in shedding of glycocalyx from the vascular endothelium, leading to higher blood concentrations.^52^, ^53^ Therefore, postoperative blood GPC1 may be affected by surgical invasion or postoperative complications. We collected postoperative samples at the first outpatient visit after discharge, but these samples might be affected by postoperative period or general conditions.

GPC1 expression in ESCC cells correlated with GPC1 protein concentration in culture medium (Figure 2A). Furthermore, the GPC1 protein concentration in the culture medium of ESCC cell lines treated with CDDP correlated with cell viability (Figure 2B). Therefore, GPC1 protein in the preoperative plasma is likely to be derived from surviving tumor cells rather than cells damaged by NAC.

In previous studies regarding the function of GPC1 in ESCC cells, suppression of GPC1 expression inhibited cell proliferation and induced cell cycle arrest at the G2/M phase.^34^ Furthermore, upregulation of GPC1 expression enhanced cell motility and induced epithelial–mesenchymal transition (EMT).^35^ Recent studies reported that an antibody‐drug conjugate targeting the GPC1 protein as an antigen demonstrated potent antitumor effects in pancreatic cancer and ESCC.^54^, ^55^ In the present study, we examined the effects of extracellular rhGPC1 on ESCC cells, and we found that extracellular rhGPC1 particularly affects cell motility, including migration, and invasion. However, in ESCC cells, extracellular rhGPC1 only increased GPC1 protein expression and had no apparent effect on GPC1 mRNA levels. It is difficult to accurately explain the mechanism by which extracellular rhGPC1 changes cellular function, but it may adhere to the cell surface or be directly uptakes into the cells. In fact, it has been reported that in cancer cells, the GCX functions as a mechanosensory organ that senses interstitial flow, and affects the expression of matrix metalloproteinase‐1 (MMP‐1), MMP‐2, CD44, and α3 integrin which regulate cell motility and metastasis induced by interstitial flow.^21^, ^56^, ^57^, ^58^, ^59^, ^60^, ^61^ Therefore, we hypothesize that extracellular GPC1 will be useful not only for an esophageal cancer progression biomarker but also as a therapeutic target for esophageal cancer.

There are several limitations to this study that should be considered. First, this study was investigated in a single‐center retrospective cohort with a small sample size. Therefore, it is difficult to reach general conclusions, and larger prospective studies will be required to establish a cutoff value for use in clinical practice. Second, the effect of endothelial cell‐derived GPC1 protein on plasma GPC1 concentrations has not been adequately considered. To address this issue, it will be necessary to collect more samples under standardized conditions in the future. Third, extracellular GPC1 may have additional effects on ESCC cells, and a more detailed analysis is required to investigate the relevance of extracellular rhGPC1 in promoting tumorigenesis and metastasis. Lastly, the concentration of extracellular rhGPC1 used in our experiments is higher than the GPC1 protein concentration in actual plasma samples, and it is debatable whether these experimental results reflect actual in vivo GPC1 function.

In conclusion, the GPC1 protein present in preoperative plasma of ESCC patients is a useful biomarker that can be analyzed as a less invasive blood sample‐based liquid biopsy. This biomarker reflects clinicopathological features, prognosis, and treatment efficacy. Lastly, we showed that extracellular GPC1 protein plays a role in both tumor cell motility and cancer progression, which indicated that it may be a candidate therapeutic target for esophageal cancer.

## AUTHOR CONTRIBUTIONS

**Rie Shibata:** Conceptualization (equal); data curation (equal); formal analysis (equal); investigation (equal); methodology (equal); project administration (equal); validation (equal); visualization (equal); writing – original draft (equal). **Hirotaka Konishi:** Project administration (equal); supervision (equal); writing – original draft (equal); writing – review and editing (equal). **Tomohiro Arita:** Methodology (equal); writing – review and editing (equal). **Yusuke Yamamoto:** Methodology (equal); writing – review and editing (equal). **Hayato Matsuda:** Methodology (equal); writing – review and editing (equal). **Taiga Yamamoto:** Methodology (equal); writing – review and editing (equal). **Takuma Ohashi:** Methodology (equal). **Hiroki Shimizu:** Methodology (equal). **Shuhei Komatsu:** Methodology (equal). **Atsushi Shiozaki:** Methodology (equal). **Takeshi Kubota:** Methodology (equal). **Hitoshi Fujiwara:** Methodology (equal). **Eigo Otsuji:** Writing – review and editing (equal).

## CONFLICT OF INTEREST STATEMENT

The authors declare no conflicts of interest.

## ETHICS STATEMENT

Approval of the research protocol by an Institutional Reviewer Board: This study was reviewed and approved by the Institutional Ethics Review Board (approval no. ERB‐C‐1414‐1).

## CONSENT

The present study was conducted in accordance with the principles of the Declaration of Helsinki and written informed consent regarding the therapy and participation in the research was obtained from all patients before surgery.

## Supporting information


Figure S1.

Figure S2.

Figure S3.

Table S1.

Table S2.

## Data Availability

The data that support the findings of this study are available on request from the corresponding author. The data are not publicly available due to privacy or ethical restrictions.
