# Supplementary figures and images for: Extracellular glypican‐1 affects tumor progression and prognosis in esophageal cancer
Source: Cancer Med. 2024 Sep 20;13(18):e70212. doi: 10.1002/cam4.70212 (PMC11413415; doi:10.1002/cam4.70212)

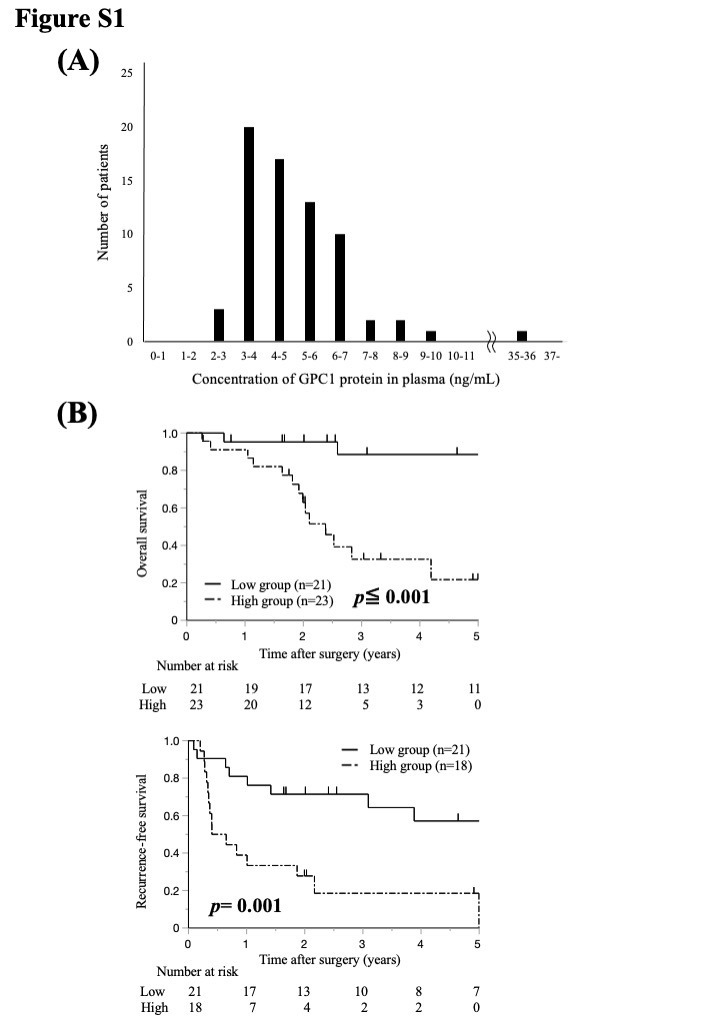

Supplement: Supplementary file 1 — Figure S1. [file CAM4-13-e70212-s004.jpg]

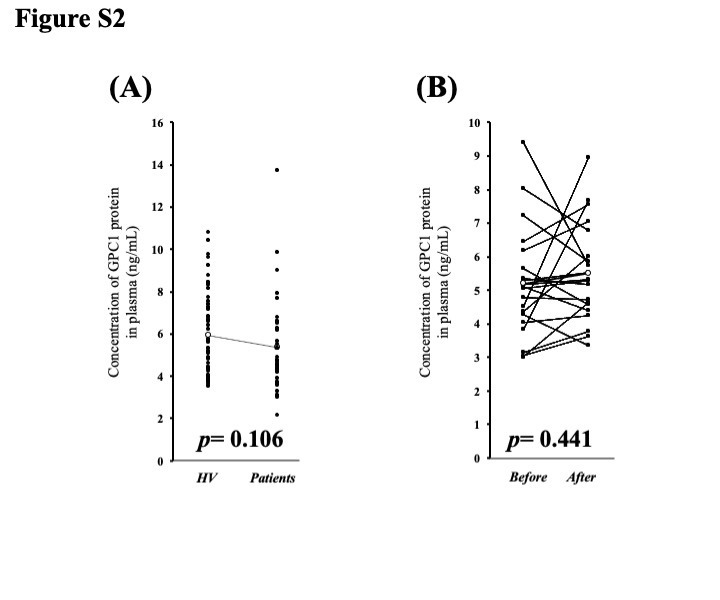

Supplement: Supplementary file 2 — Figure S2. [file CAM4-13-e70212-s005.jpg]

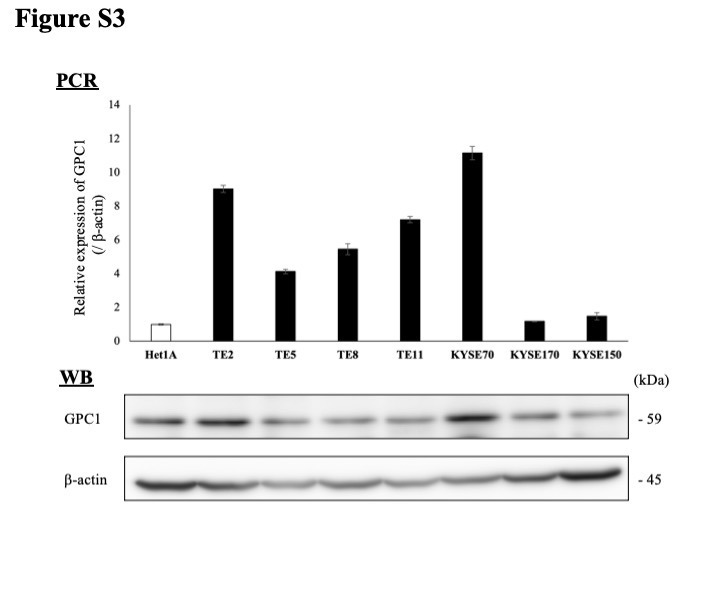

Supplement: Supplementary file 3 — Figure S3. [file CAM4-13-e70212-s001.jpg]
