# Supplementary material for: Extracellular glypican‐1 affects tumor progression and prognosis in esophageal cancer
Source: Cancer Med. 2024 Sep 20;13(18):e70212. doi: 10.1002/cam4.70212 (PMC11413415; doi:10.1002/cam4.70212)
Supplement: Supplementary file 4 — Table S1. [file CAM4-13-e70212-s003.docx]

**Table S1** Relationships between preoperative GPC1 concentration in plasma and clinicopathological factors in patients with NAC

| Variables | | *n*= 44 | Plasma GPC1 concentration | | | | *p-*value ^a^ |
| --- | --- | --- | --- | --- | --- | --- | --- |
|  |  |  | Low group (*n*= 21)  (≤ 4.67 ng/ mL) | | High group (*n*= 23)  (> 4.67 ng/ mL) | |  |
| Age (years) | ≥ 65  < 65 | 29  15 | 14  7 | (48%)  (47%) | 15  8 | (52%)  (53%) | 0.919 |
| Sex | Male  Female | 36  8 | 15  6 | (42%)  (75%) | 21  2 | (58%)  (25%) | 0.088 |
| Body mass index  (kg/m^2^) | ≥ 21  < 21 | 22  22 | 9  12 | (41%)  (55%) | 13  10 | (59%)  (45%) | 0.365 |
| PS ^b^ | 1,2  0 | 9  35 | 5  16 | (56%)  (46%) | 4  19 | (44%)  (54%) | 0.598 |
| Location | Ut  Mt  Lt | 6  22  16 | 2  10  9 | (33%)  (45%)  (56%) | 4  12  7 | (67%)  (55%)  (44%) | 0.604 |
| Differentiation | Well  Moderately  Poorly | 21  14  9 | 13  4  4 | (62%)  (29%)  (44%) | 8  10  5 | (38%)  (71%)  (56%) | 0.150 |
| Tumor size  (mm) | ≥ 30  < 30 | 30  14 | 9  12 | (30%)  (86%) | 21  2 | (70%)  (14%) | **<.001** |
| Lymphatic invasion ^c^ | Presence  Absence | 12  32 | 3  18 | (25%)  (56%) | 9  14 | (75%)  (44%) | 0.065 |
| Venous invasion ^c^ | Presence  Absence | 19  25 | 5  16 | (26%)  (64%) | 14  9 | (74%)  (36%) | **0.013** |
| pT factor ^c^ | T2,3,4  T0,1 | 27  17 | 8  13 | (30%)  (76%) | 19  4 | (70%)  (24%) | **0.003** |
| pN factor ^c^ | N1,2,3  N0 | 28  16 | 10  11 | (36%)  (69%) | 18  5 | (64%)  (31%) | **0.035** |
| pStage ^c^ | 3,4  0,1,2 | 25  19 | 8  13 | (32%)  (68%) | 17  6 | (68%)  (32%) | **0.017** |
| Residual tumor | R1,2  R0 | 5  39 | 0  21 | (0%)  (54%) | 5  18 | (100%)  (46%) | **0.023** |
| Recurrence ^d^ | +  - | 23  16 | 8  13 | (35%)  (81%) | 15  3 | (65%)  (19%) | **0.004** |
| Distant metastasis ^d^ | +  - | 12  27 | 3  18 | (25%)  (67%) | 9  9 | (75%)  (33%) | **0.016** |

ª *p-*values were calculated using the chi-squared test.

^b^ According to the Eastern Cooperative Oncology Group (ECOG) Performance Status.

^c^ According to the 8th edition of the International Union Against Cancer tumor, node, metastasis classification system.

^d^ R1 and R2 cases were excluded from the analysis.
