# Supplementary material for: Extracellular glypican‐1 affects tumor progression and prognosis in esophageal cancer
Source: Cancer Med. 2024 Sep 20;13(18):e70212. doi: 10.1002/cam4.70212 (PMC11413415; doi:10.1002/cam4.70212)
Supplement: Supplementary file 5 — Table S2. [file CAM4-13-e70212-s002.docx]

**Table S2.** Relationships between preoperative GPC1 concentration in plasma and effects of chemotherapy in patients with NAC

| Variables | | *n* = 44 | Plasma GPC1 concentration | | | | *P* value ^a^ |
| --- | --- | --- | --- | --- | --- | --- | --- |
|  |  |  | Low group (*n*= 21)  (≤ 4.67 ng/ mL) | | High group (*n*= 23)  (> 4.67 ng/ mL) | |  |
| Chemo regimen | FP  DCF  Others ^b^ | 19  23  2 | 12  9  0 | (63%)  (39%)  (0%) | 7  14  2 | (37%)  (61%)  (100%) | 0.115 |
| Clinical effect (T) ^c^ | PD  IR/SD | 0  44 | 0  21 | (-%)  (48%) | 0  23 | (-%)  (52%) | - |
| Clinical effect (N) ^c^ | SD  PR | 20  18 | 8  8 | (40%)  (44%) | 12  10 | (60%)  (56%) | 0.782 |
| Histological effect ^c^ | Grade 2/ 3  Grade 1 | 16  28 | 11  10 | (69%)  (36%) | 5  18 | (31%)  (64%) | **0.035** |
| pCR ^c^ | +  - | 7  37 | 6  15 | (86%)  (41%) | 1  22 | (14%)  (59%) | **0.028** |
| All adverse events (> g2) ^d^ | +  - | 23  16 | 11  7 | (48%)  (44%) | 12  9 | (52%)  (56%) | 0.802 |
| Neutropenia | +  - | 14  25 | 6  12 | (43%)  (48%) | 8  13 | (57%)  (52%) | 0.757 |
| Edema | +  - | 19  20 | 11  7 | (58%)  (35%) | 8  13 | (42%)  (65%) | 0.152 |
| Diarrhea | +  - | 12  27 | 4  14 | (33%)  (52%) | 8  13 | (67%)  (48%) | 0.284 |
| Fatigue | +  - | 15  24 | 6  12 | (40%)  (50%) | 9  12 | (60%)  (50%) | 0.542 |
| Stomatitis | +  - | 3  36 | 2  16 | (67%)  (44%) | 1  20 | (33%)  (56%) | 0.458 |

ª *P* values were calculated using the chi-squared test

^b^ Others : FOLFOX, mFOLFOX6

^c^ According to the Japanese Classification of Esophageal Cancer, 11th Edition

^d^ According to the Common Terminology Criteria for Adverse Events (CTCAE) version5.0

FP, Fluorouracil + Cisplatin therapy; DCF, Docetaxel + Cisplatin + Fluorouracil therapy; FOLFOX, Fluorouracil + Calcium Levofolinate Hydrate + Oxaliplatin therapy; mFOLFOX6, modified Fluorouracil + Calcium Levofolinate Hydrate + Oxaliplatin therapy; CR, complete response
